# Physical activity and risk of instant and 28-day case-fatality in myocardial infarction

**DOI:** 10.1371/journal.pone.0217398

**Published:** 2019-05-31

**Authors:** Nina Caroline Peytz, Reza Jabbari, Stig Egil Bojesen, Boerge Nordestgaard, Peter Schnohr, Eva Prescott

**Affiliations:** 1 Department of Cardiology, Bispebjerg-Frederiksberg Hospital, University of Copenhagen, Denmark, Copenhagen NV, Denmark; 2 The Copenhagen City Heart Study, Herlev Hospital, University of Copenhagen, Herlev, Denmark; Azienda Ospedaliero Universitaria Careggi, ITALY

## Abstract

**Background:**

While physical activity reduces risk of developing myocardial infarction (MI), it is unknown whether a history of physical activity is also protective of fatal arrhythmia and case-fatality in patients who have suffered an acute MI.

**Methods:**

104,801 individuals included in 2003–2014 in the Copenhagen General Population Study (CGPS), a prospective population-based study with self-reported leisure time physical activity (LTPA) in three categories measured at baseline, were followed until 2014 through national registries. The 1,517 individuals who suffered a first time MI during follow-up constituted the study population. Outcomes were fatal MI, defined as date of death same as date of MI (including out-of-hospital deaths) and 28-day fatality. Through multivariable analyses the association between baseline LTPA and outcomes were assessed adjusted for CVD risk factors.

**Results:**

Of 1,517 MI events, 117 (7.7%) were fatal and another 79 (5.6%) lead to death within 28 days. Median time from baseline to MI was 3.6 years (IQR 1.7–5.8). LTPA was associated with lower risk of fatal MI with odds ratios of 0.40 (95% CI: 0.22–0.73) for light and 0.41 (0.22–0.76) for moderate/high LTPA after multivariable adjustment with sedentary LTPA as reference. Age, alcohol-intake, education and smoking were identified as other predictors for fatal MI. We found no association between LTPA and 28-day case fatality.

**Conclusions:**

Among individuals with MI, those that have engaged in any light or moderate physical activity were more likely to survive their MI. Results are consistent with effect of exercise preconditioning on risk of fatal arrhythmia.

## Introduction

Coronary artery disease (CAD) and its consequence, myocardial infarction (MI), are believed to underlie 75% of the deaths of patients who experience sudden cardiac death (SCD) [1]. The greatest risk of SCD in acute MI is in the initial phase after which the risk declines rapidly [2]. It has been estimated that at least two thirds of fatal coronary events occur before the patient reaches a hospital [3]. As a consequence primary prevention is essential to lowering rates of fatal MI.

Regular physical activity is associated with decreased risk of fatal CAD and SCD [4]. The cardioprotective effect has been documented in numerous studies for more than 30 years [5]. It is plausible that in addition to preventing MI, the physical activity prior to MI could modify the course of MI. Strenuous physical activity may trigger acute MI and cardiac arrhythmias with subsequent risk of cardiac death [6,7], while physical activity with moderate intensity is correlated with reduced risk of cardiac arrest [8]. Multiple experimental models [9,10] have demonstrated the ability of exercise to induce ischemic preconditioning, referring to the capacity of brief episodes of myocardial ischemia to protect against cellular damage during subsequent sustained periods of ischemia. A recent review has suggested that clinically relevant cardioprotection through preconditioning can be obtained after just a single episode of exercise [11]. Exercise preconditioning has the potential to reduce case fatality in the initial stages of MI. While the preconditioning effect of exercise is well proven in animal models [9,12], studies in humans are lacking. Only a few studies in humans have compared risk factors leading to fatal versus non-fatal MI in study populations that include out-of-hospital SCD [13–15] and only one has addressed physical activity [15]. In a population-based study we recently demonstrated that self-reported regular physical activity was associated with a 45% lower case fatality in MI with a dose-response association between increasing level of physical activity and decreased same-day fatality [15]. However, baseline information was gathered up to several decades before the cardiac event and may have changed over the time elapsed. We therefore addressed this question in a larger and more recent prospective population-based cohort with shorter time-span between baseline examination and incident MI.

This study utilizes data from the Copenhagen General Population Study (CGPS) to assess level of leisure-time physical activity (LTPA) as a predictor for fatal MI and subsequent 28-day case fatality in MI adjusting for known CAD risk factors to further clarify whether exercise preconditioning in humans may lead to cardioprotection in MI.

## Methods

### Study population

This study is based on 104,801 individuals of Danish descent aged 20–100+ years from the Copenhagen General Population Study (CGPS), a prospective epidemiological cohort study with participants randomly selected from the Danish Civil Registration System living in a defined area around Copenhagen. Recruitment began in 2003 and is ongoing with a response rate of those invited around 45% [16]. The study was approved by The Committee on Biomedical Research Ethics for the Capital Region in Denmark (#H-KF-01-144/01). Written informed consent was obtained from all participants.

The present study includes all individuals in the CGPS who presented with acute MI (International Classification of Diseases 10^th^ revision codes I21-I22 [17]). Individuals with MI or stroke prior to inclusion identified through registries and self-report were excluded. In addition, those with missing or incomplete information regarding leisure-time physical activity (LTPA), body mass index (BMI), blood pressure, cholesterol and education were excluded.

### Study outcome and follow-up

The primary outcome of the present study was fatal MI defined as date of death being the same as the date of the acute MI including any out-of-hospital death registered with MI as cause. The secondary outcome was 28-day fatality in MI, defined as death within 28 days of the acute MI excluding those who died on the same date as their MI.

Participants were followed from their examination until death or the 14^th^ of November 2014, whichever came first, for fatal and non-fatal MI as well as all-cause mortality obtained from the Danish Cause of Death Register, the National Hospital Discharge Register and the Danish Civil Registration System. Through these registers any diagnosis of acute MI was available as well as cause of death including cause of death for any pre-hospital deaths. There was no loss to follow-up except through emigration. Those who emigrated were censored at the date of emigration [18].

### Baseline variables

All participants in CGPS completed a comprehensive questionnaire at inclusion detailing lifestyle and medical history. A health examiner performed a physical examination including height, weight and resting blood pressure. A nonfasting blood sample was drawn.

The primary exposure variable leisure-time physical activity (LTPA) was assessed by the Copenhagen General Population Study LTPA questionnaire [19,20]. Participants graded their LTPA in the last year as belonging to one of four categories: *Sedentary*: Almost completely inactive or light activity less than 2 hours per week (e.g. reading, television and cinema). *Light activity*: Light physical activity 2–4 hours per week (e.g. walking, bicycling, light gardening and light gymnastics). *Moderate activity*: Light activity more than 4 hours per week or more strenuous physical activity 2–4 hours per week (e.g. brisk walking, fast bicycling, heavy gardening and strenuous gymnastics). *High activity*: More strenuous or physical activity more than 4 hours per week, regular hard training or competitive sport multiple times per week. The groups with moderate and high level of LTPA were merged into one to ensure adequate group sizing.

The following variables were considered as potential confounders: Age at MI, sex, body mass index (BMI), self-reported diabetes, systolic blood pressure, plasma total cholesterol, weekly alcohol intake, education, and smoking status (current smoker, former smoker or never smoker). As income may not reflect socioeconomic status in the elderly, education was selected as the indicator of socioeconomic status. Education was stratified into two groups: 0–9 years corresponding to primary school and >9 years corresponding to any level of secondary schooling.

BMI was measured as weight divided by height squared (kg/m^2^) and was stratified for analysis into three groups: normal (BMI<25), overweight (BMI 25–29.9) and obese (BMI > = 30) as defined by the WHO classification [21]. Alcohol intake was self-reported as number of standard drink units per week, with one unit defined as 12 gram of alcohol. Alcohol intake was stratified into two groups: 0–7 units/week and >7 units/week.

### Statistical analysis

Participants were followed from their examination until death, emigration or the 14^th^ of November 2014 whichever came first. The main outcome was fatal MI. Stratified by LTPA level or MI survival status, the baseline information of individuals were compared across strata using one-way analysis of variance (ANOVA) for continuous normally distributed variables, Kruskal-Wallis test for continuous skewed variables and Pearson’s chi-squared for categorical variables.

To identify predictors of immediate fatal MI and 28-day MI case fatality both univariable and multivariable logistic regression analysis were performed for each outcome to calculate odds ratios (OR) for case fatality in MI. Multivariable logistic models included traditional cardiovascular risk factors of age, sex, BMI, diabetes, systolic blood pressure, total cholesterol, alcohol intake, education and smoking status as independent variables as well as LTPA. By backwards elimination any variable that did not contribute to the multivariable model with a P-value < 0.1 was removed.

The statistical analysis was carried out using Stata version 13.

## Results

### Baseline data

**Fig 1:**
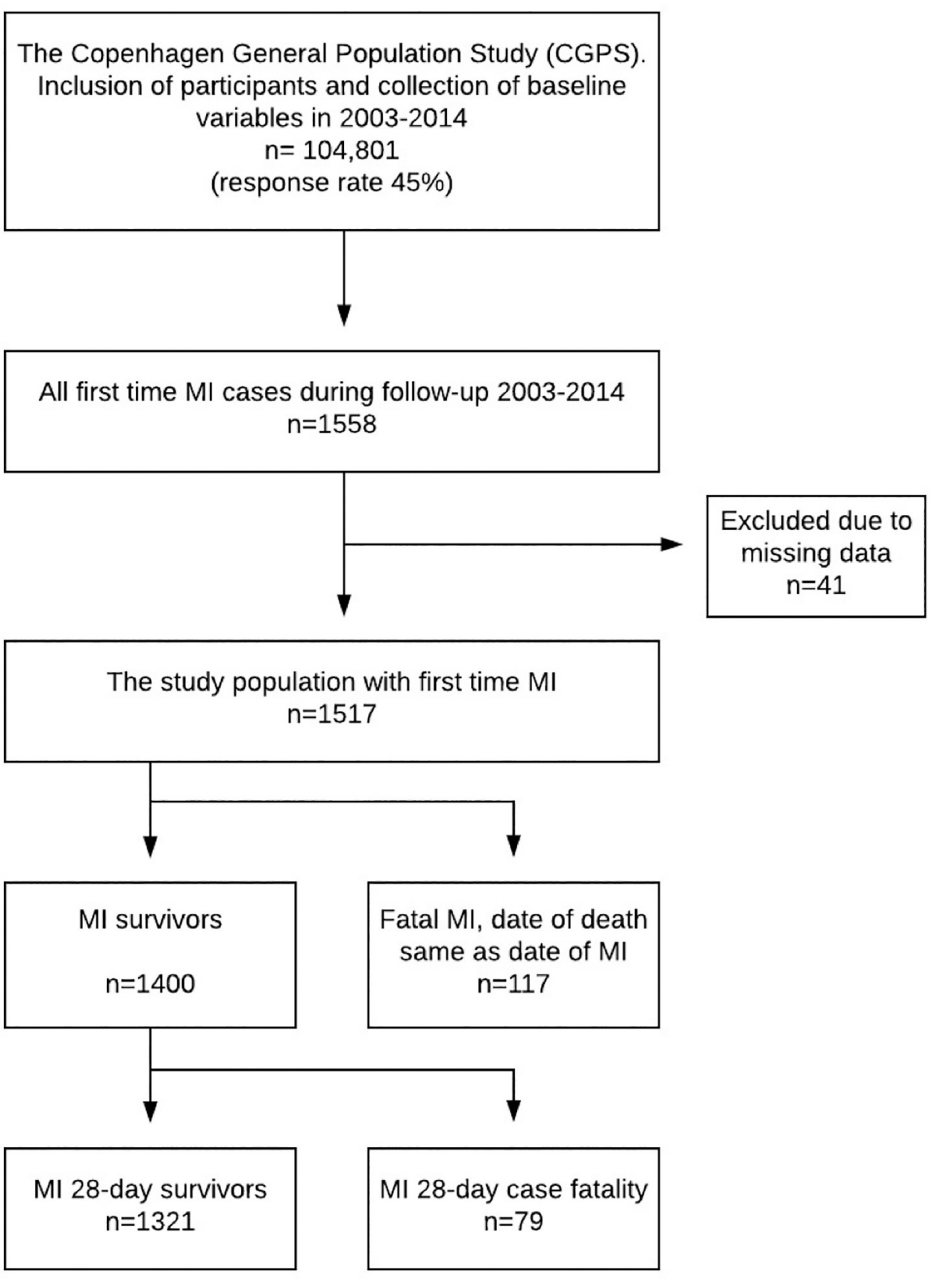
Study cohort. Flowchart of the study cohort with follow-up of first time myocardial infarction (MI) from the Copenhagen General Population Study from 2003 to 2014.

A total of 1,558 participants from the Copenhagen General Population Study presented with first time MI during follow-up. After excluding 41 participants because of incomplete information, the study population consisted of 1517 participants (Fig 1).

Of the 1,517 individuals with first time MI, 951 (62.7%) were men, mean age at MI was 70.8 years, and median time from baseline to MI diagnosis was 3.6 years (Table 1). The majority of the individuals with MI had reported to practice either light (50.5%) or moderate/high 41.3%) LTPA. The individuals reporting to be sedentary were younger, had a higher BMI, higher prevalence of diabetes, were more likely to smoke, reported lower alcohol consumption and had shorter time from inclusion to incident MI.

**Table 1:** Baseline characteristics of the study population with myocardial infarction (MI) stratified by level of leisure-time physical activity (LTPA) level, Copenhagen General Population Study 2003–2014.

	All	Sedentary	Light LTPA	Moderate/high LTPA	P value
	N = 1,517	N = 125	N = 766	N = 626	
	(100%)	(8.2%)	(50.5%)	(41.3%)	
Age at MI, mean (SD), years	70.8 (11.6)	67.6 (13.1)	71.6 (11.6)	70.4 (11.1)	**0.001**
Time baseline-MI, median (IQR), years	3.6 (1.7–5.8)	2.9 (1.1–5.0)	3.6 (1.7–5.9)	3.7 (1.8–5.9)	**0.03**
Male (%)	951 (62.7)	77 (61.6)	432 (56.4)	442 (70.6)	**<0.001**
BMI, median (IQR), kg/m^2^	27.0 (24.5–29.9)	28.1 (24.7–32.4)	27.1 (24.6–30.1)	26.7 (24.5, 29.1)	**<0.001**
Diabetes (%)	202 (13.3)	34 (27.2)	103 (13.4)	65 (10.4)	**<0.001**
Systolic blood pressure, mean (SD), mmHg	150.1 (20.7)	148.8 (21.1)	150.0 (20.6)	150.5 (20.9)	0.69
Total cholesterol, mean (SD), mg/dl	224 (46)	224 (50)	224 (46)	228 (46)	0.64
Alcohol intake >7 units/week^a^ (%)	838 (55.2)	57 (45.6)	400 (52.2)	381 (60.9)	**0.002**
Education (%)					0.15
0–9 years	752 (49.57)	58 (54.4)	391 (51.0)	293 (46.8)	
>9 years	765 (50.43)	57 (45.6)	375 (49.0)	333 (53.2)	
Smoking status^a^ (%)					**<0.001**
Never	406 (26.8)	19 (15.2)	189 (24.7)	198 (31.6)	
Current	410 (27.0)	63 (50.4)	197 (25.7)	150 (24.0)	
Former smoker	697 (45.9)	43 (34.4)	377 (49.2)	277 (44.2)	
**Outcome**					
Fatal MI (%)	117 (7.7)	18 (14.4)	55 (7.2)	44 (7.0)	**0.014**
28-day case fatality for non-fatal MI (%)	79 (5.6)	7 (6.5)	42 (5.9)	30 (5.2)	0.77

Data is presented as means (SD) for normal distributed continuous variables and number (%) for categorical variables. Time from baseline to MI and BMI are presented as median (25^th^-75^th^percentiles).

^a^Missing data, therefore total is not 1,517.

### LTPA and risk of fatal MI

A total of 117 (7.7%) of the 1517 MI cases were fatal within the same day. There was no significant difference in time from baseline to incident MI between fatal and non-fatal events (p = 0.14) (Table 2). Those presenting with fatal MI were more likely to be older, have fewer years of education and report a lower LTPA level.

**Table 2:** Baseline characteristics of the study population with myocardial infarction (MI) stratified by survival status of MI and 28-day case fatality, Copenhagen General Population Study 2003–2014.

	Fatal MI	Non-fatal MI	P value	28-day fatality	28-day survival	P value
	N = 117	N = 1,400		N = 79	N = 1,321	
	(7.7%)	(92.3%)		(5.6%)	(94.4%)	
Age at MI, years	76.4 (9.7)	70.3 (11.6)	**<0.001**	80.0 (10.0)	69.7 (11.4)	**<0.001**
Time baseline-MI, years	3.3 (1.3–5.6)	3.6 (1.8, 5.9)	0.14	4.2 (2.2, 6.4)	3.6 (1.7, 5.8)	0.07
Male	76 (65.0)	875 (62.5)	0.6	49 (62.0)	826 (62.5)	0.93
BMI, kg/m^2^	26.7 (24.1, 29.8)	27.0 (24.5,29.9)	0.48	27.0 (24.6, 29.9)	27.0 (23.2, 29.2)	0.29
Diabetes	18 (13.1)	184 (15.4)	0.49	13 (16.5)	171 (12.9)	0.37
Systolic blood pressure, mmHg	150.1 (18.6)	150.1 (20.9)	0.51	153.2 (23.2)	149.9 (20.8)	0.91
Total cholesterol, mg/dl	220 (42)	228 (46)	0.09	220 (46)	228 (46)	0.11
Alcohol intake >7units/week^a^	75 (64.1)	763 (54.5)	0.08	34 (49.4)	724 (54.8)	0.57
Education			0.054			0.93
0–9 years	68 (58.1)	684 (48.9)		39 (49.4)	645 (48.8)	
>9 years	49 (41.9)	716 (51.1)		40 (50.6)	676 (51.2)	
Smoking status^a^			0.13			0.2
Never	22 (18.8)	384 (27.4)		20 (25.3)	364 (27.6)	
Current	36 (30.8)	374 (26.7)		20 (25.3)	354 (26.8)	
Former smoker	68 (49.6)	639 (45.6)		38 (48.1)	601 (45.5)	
LTPA			**0.014**			0.77
Sedentary	18 (15.4)	107 (7.6)		7 (8.9)	100 (7.6)	
Light	55 (47.0)	711 (50.8)		42 (53.2)	669 (50.6)	
Moderate/high	44 (37.6)	582 (41.6)		30 (38.0)	552 (41.8)	

Data is presented as means (SD) for normal distributed continuous variables and number (%) for categorical variables. Time from baseline to MI and BMI are presented as median (25^th^-75^th^percentiles).

^a^Missing data, therefore total is not 1,517.

By univariable logistic regression a sedentary LTPA level was identified as associated with fatal MI, as were older age, alcohol intake >7 units/week and lower level of education (Table 3). In the multivariable logistic model, the association was strengthened: With a sedentary LTPA level as reference point a light LTPA level had an OR of 0.40 (95% CI 0.22–0.73) and moderate/high LTPA level had an OR of 0.41 (0.22–0.76). There was no significant difference in odds of fatal MI between light and moderate/high baseline level of LTPA and thus, no dose-dependent association was apparent.

**Table 3:** Predictors of immediate MI case fatality. Univariable and multivariable logistic regression analysis of incident fatal MI (defined as death on the same date as MI).

	Crude	Multivariable^a^
Age at cardiac event	**1.05 (1.03–1.07)**	**1.06 (1.04–1.08)**
Male	1.11 (0.75–1.65)	-
BMI	1.03 (0.67–1.60)	-
<25	Reference	-
25–29.9	0.88 (0.57–1.37)	-
> = 30	0.96 (0.58–1.59)	-
Diabetes type I or II	1.20 (0.71–2.03)	-
Systolic blood pressure	1.00 (0.99–1.01)	-
Total cholesterol	0.90 (0.75–1.05)	-
Alcohol-intake >7 units week	**1.57 (1.03–2.38)**	**1.73 (1.12–2.68)**
Years of education		
0–9	Reference	-
>9	0.69 (0.47–1.01)	-
Smoking status		
Never smoker	Reference	Reference
Current smoker	1.68 (0.97–2.90)	**1.87 (1.05–3.31)**
Former smoker	1.58 (0.95–2.62)	1.40 (0.83–2.35)
LTPA level		
Sedentary	Reference	Reference
Light	**0.46 (0.26–0.81)**	**0.40 (0.22–0.72)**
Moderate/High	**0.45 (0.25–0.80)**	**0.41 (0.22–0.75)**

Data is presented as Odds Ratio (95% confidence interval).

^a^Multivariable model including age, sex, BMI, diabetes, systolic blood pressure, cholesterol, alcohol intake, education, smoking status and level of leisure-time physical activity.

### LTPA and 28-day MI case fatality

Of the 1,400 individuals who survived their MI initially, 79 (5.6%) died within 28 days. The survivors were younger with a mean age of 67.9 years while the non-survivors had a mean age of 80.0 (P <0.001). In univariable and multivariable logistic regression models adjusting for known CAD risk factors, only age was found as associated with 28-day MI case fatality with an OR of 1.10 (95% CI 1.07–1.13) (Table 4). Adjusted for age with light LTPA as reference, the OR was 1.49 (95% CI 0.62–3.56) for sedentary level and 1.00 (0.61–1.64) for moderate/high LTPA level.

**Table 4:** Predictors of 28-day MI case fatality. Univariable and multivariable logistic regression analysis of incident MI with death within 1–28 days of cardiac event.

	Crude	Multivariable^a^
Age at cardiac event	**1.10 (1.07–1.13)**	**1.10 (1.07–1.13)**
Male	0.98 (0.61–1.56)	-
BMI	0.85 (0.49–1.49)	-
<25	Reference	-
25–29.9	0.88 (0.52–1.47)	-
> = 30	0.79 (0.42–1.48)	-
Diabetes type I or II	1.32 (0.72–2.45)	-
Systolic blood pressure	1.01 (0.99–1.02)	-
Total cholesterol	0.88 (0.72–1.08)	-
Alcohol-intake >7 units week	0.83 (0.52–1.33)	-
Years of education		
0–9	Reference	-
>9	0.98 (0.62–1.54)	-
Smoking status		
Never smoker	Reference	-
Current smoker	1.03 (0.54–1.94)	-
Former smoker	1.15 (0.66–2.01)	-
LTPA level		
Sedentary	1.12 (0.49–2.55)	1.49 (0.62–3.56)
Light	Reference	Reference
Moderate/High	0.87 (0.53–1.40)	1.00 (0.61–1.64)

Data is presented as Odds Ratio (95% confidence interval).

^a^ Multivariable model including age, sex, BMI, diabetes, systolic blood pressure, cholesterol, alcohol intake, education, smoking status and level of leisure-time physical activity

## Discussion

In this prospective population-based study the main finding was that subjects with MI who were sedentary prior to their cardiac event were more likely to suffer an immediately fatal event. There seemed to be no further cardioprotection with increasing level of physical activity. 28-day MI case fatality was not associated with level of physical activity, but the 28-day case fatality was low.

This study expands the knowledge on the association between physical activity and MI mortality risk. Our results suggest that the risk factors for fatal versus non-fatal MI differ. As most previous studies group have either grouped fatal and non-fatal MI together or have not included fatal MI occurring outside hospital settings, the relatively greater importance of some risk factors for fatal MI has not been appreciated.

### Physical activity and fatal MI

The current study identified being sedentary as associated with fatal MI. Subjects reporting a sedentary LTPA level had a more adverse baseline CAD risk factor profile than those reporting higher LTPA levels. Nevertheless, even when adjusting for these variables, individuals with light and moderate/high LTPA had almost 60% lower risk of immediate case fatality. Age, smoking, and alcohol consumption were also associated with fatal MI. A Swedish nested case-control study of predictors for SCD in MI including out-of-hospital deaths, identified low education, diabetes and higher BMI as contributing to increased risk of 24-hour case fatality, but did not include information on physical activity [13].

In the present study higher levels of physical activity did not appear more beneficial than light. This is in contrast to our previous study, where we found a dose-dependent association between LTPA level and fatal MI with ORs of 0.70 (95% CI 0.53–0.92) and 0.55 (0.39–0.76) for light and moderate/high LTPA, respectively [15]. A retrospective review of the impact of LTPA on primary cardiac arrest also found that moderate intensity activity was similar to that of high intensity activity in reducing risk [8]. In this study moderate-intensity activity was comparable to the light LTPA level defined in the present study. This lends support to the findings of the present study and may suggest that a beneficial effect of LTPA on SCD rates does not increase linearly with increasing activity.

### Comparing case-fatality in MI

The current study found that immediate MI case fatality was 7.7%, a number that is quite low in comparison to earlier studies of fatal MI. In data from another Danish population study, the Copenhagen City Heart Study, 25.5% of MI cases in 1978–2013 were immediately fatal [15]. Another prospective cohort study from 1986–2006 reported a 24-hour MI case fatality around 15% [13]. With improvement in acute care and preventive treatment, there has been a steep decline in MI case fatality [22,23]. The lower incidence of fatal MI in the present study may be more representative of the current risk.

The current study investigated fatal MIs including any out-of-hospital cases registered in national registries with MI as cause of death. It is worth noting that cause-of-death registration entails some inaccuracy [24]. A study investigating the validity of the acute MI diagnosis in Danish national registries found that the sensitivity was lower for mortality data in non-hospitalized patients but confirmed the use of registries as a valid tool in population-based cohorts [25]. That study noted that inclusion of deaths with MI as a secondary diagnosis or contributory cause of death increased the sensitivity greatly with only a slight decrease in predictive value. It is thus likely that we have underestimated case-fatality of MI. This, however, is a challenge for all population-based studies relying on cause-of-death registries. Though non-hospitalized fatal cases may be subject to some inaccuracy, the inclusion of these cases is crucial when studying fatal MI as most occur outside hospital settings [3]. It is not likely that the inaccuracy is associated with LTPA and thus not likely to significantly bias the results.

### Possible mechanisms of lower case fatality

Although a beneficial effect of physical exercise on CAD development and subsequent mortality has been extensively documented in the medical literature the mechanisms through which the cardioprotective effect is obtained are not fully understood. Some effect is likely to be through risk factor modification [26–29]. Animal studies have repeatedly demonstrated that exercise can reproduce ischemic preconditioning with subsequent decrease in infarction size [30,31]. Preconditioning by exercise offers an immediate cardioprotective effect as discussed in a recent review [11]. A case-control study investigating risk factors for ventricular fibrillation in acute MI patients found preinfarction-angina to be inversely associated with ventricular fibrillation after multivariable adjustment [32]. Preinfarction-angina is a known surrogate for ischemic preconditioning, and it is plausible that ischemic preconditioning by physical activity may have a similar effect.

Previous investigations of exercise and preconditioning in humans has focused on preconditioning through exercise-induced ischemia [31], while no experimental studies to our knowledge have investigated the effect of regular physical activity in human models. An animal study suggested that the intensity of exercise does not alter the magnitude of cardioprotection [33], while another suggested that even low intensity exercise have the ability to mediate greater recovery of cardiac output after myocardial ischemia [34]. This is supported by the findings of the present study.

### 28-day MI case fatality

The present study did not identify any protective effect associated with LTPA on the MI 28-day case fatality. This is consistent with our previous study in which physical activity was associated with lower instant MI fatality but not with subsequent risk of all-cause mortality [15]. These findings may reflect different mechanisms of death in the acute and the subsequent stages of a MI. However, it must be noted that our study had relatively few outcomes, as reflected in the wide confidence intervals (Table 4). Sedentarism cannot be ruled out as a risk factor for 28-day MI case fatality.

Previous studies of the association between physical activity and MI case fatality have identified a number of clinical findings as predictors of prognosis but most have not included out-of-hospital deaths and have not distinguished between predictors of immediate mortality and mortality in the subsequent stages [35]. Our findings stress the importance of making this distinction in future studies of CAD case fatality.

### Strengths and limitations

To the best of our knowledge, this is one of the first studies utilizing a large prospective population-based cohort to investigate the association between LTPA and MI case fatality including pre-hospital MI case fatalities, while distinguishing between immediate fatal MI and subsequent 28-day case fatality.

Our findings imply that different mechanisms may contribute to the mortality in the acute setting and the following month. The study carries substantial strength by its design and inclusion of prospectively recorded information, permitting adjustment for numerous possible confounders. The use of national registers with minimal loss of follow-up contributes to minimize attrition bias. Median time from baseline to MI was 3.6 years, which is low compared to similar studies, and the baseline information on LTPA and other risk factors is likely to be valid at MI onset for most individuals. A population-based study with repeated assessment of physical activity found strong correlation between physical activity reported at baseline and at re-survey three years later [36].

All patients with previous MI or stroke were excluded to reduce risk of self-selection bias. Though the analyses were adjusted for multiple possible confounders, our study did not adjust for additional comorbidities. While reverse causation represent an important concern, a recent study of the Copenhagen City Heart Study found this to be negligible in the association between LTPA and CAD mortality [37]. The findings regarding this association remained unchanged after adjusting for occurrence of CAD and cancer during follow-up. As the study design of the CGPS is similar one may conclude that this relationship extends to our study.

A limitation of the current study is that level of LTPA was self-reported as belonging to one of four broad groups. These groups, however, have previously been found to carry distinct excess risk of CAD outcomes and all-cause mortality [20,38]. Our study included recent MI cases in 2003–2014, which is likely to be representative of current MI populations.

In conclusion, physical activity was identified to be independently associated with lower MI case fatality, but the mortality risk did not decrease with increasing activity level. The results are consistent with a preconditioning effect of exercise on cardioprotection in MI.
